# Overnight admissions to a neonatal intensive care unit in Ethiopia are not associated with increased mortality

**DOI:** 10.1371/journal.pone.0264926

**Published:** 2022-03-24

**Authors:** Rishi P. Mediratta, Mallika Rajamani, Mulugeta Ayalew, Abdulkadir Shehibo, Ashenafi Tazebew, Alemayehu Teklu

**Affiliations:** 1 Division of Pediatric Hospital Medicine, Department of Pediatrics, Stanford University School of Medicine, Stanford, California, United States of America; 2 College of Medicine, SUNY Upstate Medical University, Syracuse, New York, United States of America; 3 Department of Pediatrics and Child Health, College of Medicine and Health Sciences, University of Gondar, Gondar, Ethiopia; University of Mississippi Medical Center, UNITED STATES

## Abstract

**Background:**

In 2019, 2.4 million neonates died globally, with most deaths occurring in low-resource settings. Despite the introduction of neonatal intensive care units (NICUs) in these settings, neonatal mortality remains high, and caring for sick neonates around the clock can be challenging due to limited staff and resources.

**Objective:**

To evaluate whether neonatal intensive care admissions during daytime and overnight hours affects in-hospital neonatal mortality.

**Methods:**

A retrospective case-control study was conducted using 2016 chart data at a University hospital in Ethiopia. Cases were defined as neonates who died in the NICU, and controls were defined as neonates who survived. Overnight hours were defined as 17:00 to 07:59, and day hours were defined as 08:00 to 16:59. Univariate and multivariate logistic regressions were used to investigate the relationship between time of admission and mortality, along with perinatal characteristics.

**Results:**

A total of 812 neonates, 207 cases and 605 controls, met inclusion criteria. There were 342 admissions during the day and 470 overnight. Neonatal mortality (aOR 1.02, 95% CI [0.64–1.62], p = 0.93) was not associated with overnight admissions after controlling for maternal age, parity, C-section, birthweight, and gestational age, respiratory distress, and admission level of consciousness. Admission heart rate >160 (aOR 0.52, 95% CI [0.30–0.91], p = 0.02) was the only variable significantly associated with overnight admissions.

**Conclusion:**

Being admitted overnight to the NICU in Gondar, Ethiopia was not associated with increased mortality, consistent with a constant level of care, regardless of the time of admission. Further qualitative and implementation research are needed to understand contextual factors that have affected these data.

## Introduction

An estimated 2.4 million neonates under 28 days of life die every year [1]. Global efforts have reduced the neonatal mortality rate (NMR) by 47% since 1990 [1]. Neonatal intensive care units (NICUs) have contributed to the improved survival of neonates. Specific training on neonatal resuscitation and the use of medical technology for neonates in NICUs has helped reduce neonatal mortality in high-income countries [2]. Nonetheless, the neonatal mortality rate in low- and middle- income countries (LMICs) remains unacceptably high. High quality critical care services should be available for all neonates, yet NICUs in LMICs lack essential expertise, medications, and equipment [3–7]. This has contributed to the significant disparities in the rates of neonatal mortality across regions and countries [8].

Ethiopia is no exception to the high level of neonatal mortality seen in LMICs. In 2019, the country had a reported NMR of 28 deaths per 1,000 live births, commensurate with the NMR across Sub-Saharan Africa [9]. Though this rate has been steadily decreasing over the past two decades, it has declined more slowly compared to mortality rates among infants and children under five [1]. To address this, there has been a recent push in Ethiopia to improve care in NICUs across the country [10]. Despite these improvements, however, a neonate born in Sub-Saharan Africa is ten times more likely to die within the first month of life than a neonate born in a high-income country [1].

There are known circadian variations with NICU admissions and mortality in high-income countries. Researchers in Canada found that premature neonates who are admitted to the NICU during overnight hours may have a higher risk of mortality, after controlling for patient acuity [11]. Conversely, premature infants admitted overnight to a network of NICUs in Australia did not have an increase in neonatal mortality [12]. The association between the time of NICU admission and neonatal mortality has not been studied in low-income countries. Circadian differences in neonatal mortality by time of admission has the potential to influence the organization and delivery of healthcare for millions of neonates globally who are admitted to NICUs overnight.

We investigated the association between time of NICU admission and neonatal mortality by comparing daytime with overnight admissions in a tertiary care NICU in Ethiopia. We hypothesized that neonates admitted to the NICU overnight would have increased mortality compared to those admitted during the day given the documented lack of high-quality critical care services in many LMICs.

## Methods

### Study design

This study was a retrospective case-control study of prospectively collected data. Patients were recruited from the NICU registry from January 1, 2016 to December 31, 2016. The study was reviewed and exempt from the institutional review board (IRB) at the Stanford University School of Medicine and the University of Gondar Hospital IRB. Data were extracted between 4/2018 to 6/2018. No personal identifiers were recorded to maintain anonymity and confidentiality of participants. Medical records were accessed with permission of the University of Gondar Hospital and the University of Gondar IRB. The need for written informed consent from patients was waived because the retrospective nature of the study, and this study was part of a hospital-based quality improvement project and posed no risk to patients or their privacy. Patient records were anonymized and de-identified prior to analysis.

### Study setting

The University of Gondar is in the Amhara region of Ethiopia, approximately 700 km from the capital, Addis Ababa. It is one of the largest referral and academic hospitals in the country, providing care to over 7 million people and approximately 10,000 children every year. The NICU in Gondar is a 40-bed unit that has the capacity for phototherapy, nasogastric tube feedings, thermoregulation, IV fluids, blood transfusions, oxygen via nasal cannula, and bubble continuous positive airway pressure (CPAP). The NICU admission criteria include the following: birthweight less than 2000 grams, gestational age less than 34 weeks, suspected or confirmed infection, temperature instability, respiratory distress, apnea, cyanosis, electrolyte derangements, birth trauma, seizures, birth asphyxia, altered mentation, feeding problem, bilious emesis, signs of bowel obstruction, hyperbilirubinemia, ABO and Rh incompatibility, anemia, polycythemia, bleeding disorder, cardiovascular disease requiring monitoring or interventions, any baby whom the physician or nurse feels requires observation or treatment, and social issues like abandoned babies [13]. There is no neonatologist; rather, the NICU is staffed by nurses, sixth-year medical students, pediatric residents, and general practitioners, and supervised by an attending pediatrician. During the day, there are approximately 12 interns, 5 residents, and 12–15 nurses in the NICU. Overnight, the NICU is staffed by approximately 5 interns, 2 residents, and 6–8 nurses.

### Patient selection

Cases were defined as neonates who died in the NICU, and controls were defined as neonates who survived. Patients older than 28 days, outside of the accrual period, and revisits were excluded. We recruited approximately three times as many controls as cases. Data abstracters were not blind to the predictors or the outcome.

### Outcome variable

The main outcome variable was daytime versus overnight admission. Daytime admission was defined as NICU admission occurring between 08:00 and 16:59. Overnight admission was defined as NICU admission between 17:00 and 07:59 the following day, corresponding to the time house staff in the NICU are on call.

### Predictor variables

The following predictor variables were extracted from paper charts of neonates based on review of the literature, clinical usefulness, and biological plausibility: age at admission, gender, gestational age, type of delivery, duration of labor, duration of rupture of membranes, APGAR scores, birth weight, head circumference, and length of the baby. Clinical values included admission heart rate, respiratory rate, temperature, mental status, and respiratory distress.

Vital signs upon NICU admission were extracted as a continuous variable. Temperature and birth weight were classified according to WHO definitions [14]. Normal respiratory rates and heart rates were defined according to established ranges [15]. Respiratory distress was categorized as none; mild distress had subcostal and intercostal retractions; moderate distress had grunting, subcostal, intercostal, and nasal flaring; severe distress had grunting, subcostal, intercostal, and nasal flaring with perioral cyanosis. Since routine prenatal ultrasounds are not routinely available, the gestational age of each infant was determined using the New Ballard score.

Small-for-gestational age (SGA), appropriate-for-gestational age (AGA), large-for-gestational age (LGA), microcephalic, normocephalic, and macrocephalic were defined according to the reference distributions for gender and birthweight [16]. SGA was defined as birthweight below the 10^th^ percentile for gestational age, AGA was defined as birthweight between the 10^th^ and 90^th^ percentiles for gestational age, and LGA was defined as birthweight above the 90^th^ percentile for gestational age. Microcephalic was defined as head circumferences below the 10^th^ percentile for gestational age, normocephalic was defined as head circumference between the 10^th^ and 90^th^ percentiles for gestational age, and macrocephalic was defined as head circumference greater than 90^th^ percentile for gestational age.

Case mix, or severity of illness upon NICU admission, was assessed using the Neonatal Mortality Score (NMS), a validated measure of NICU mortality risk designed specifically for LMICs and derived from the study population [13]. The score ranges from 0 to 52, and incorporates admission level of consciousness, admission respiratory distress, gestational age, and birthweight (Table 1). Scores greater than 12 correspond to a 50% probability of in-hospital mortality [13].

**Table 1:** Neonatal mortality score.

Characteristic	Score
Admissions Level of Consciousness	
Alert	0
Irritable	6
Lethargic	11
Comatose	16
Admission Respiratory Distress	
None	0
Mild (subcostal and intercostal retractions)	3
Moderate (subcostal and intercostal retractions, nasal flaring, and grunting)	11
Severe (subcostal and intercostal retractions, nasal flaring, grunting, and perioral cyanosis)	14
Gestational Age	
≥ 37 weeks	0
32–36 weeks	1
<32 weeks	10
Birthweight	
≥2500 grams	0
1500–2499 grams	5
<1500 grams	12
Maximum Score	52

### Sample size

No prior estimates were available that could be used to calculate the sample size for the study. Hence, the rule of thumb of 10 events per variable for logistic regression prediction models was used to estimate the sample size [17]. Since there were approximately 20 candidate variables considered and 10 events per variable, the estimated number of cases for the study was 200.

### Missing data

Prediction variables missing 15% or more of data were excluded from the analysis. Missing categorical data were imputed with the mode, and missing continuous data were imputed with the median.

### Statistical analysis

We conducted a descriptive analysis to compare characteristics of admitted neonates during daytime and overnight hours by computing the frequencies and percentages for data. Unadjusted time from NICU admission to death or discharge was evaluated using a Kaplan-Meier curve. Differences in the in-hospital mortality between neonates admitted during daytime and overnight shifts were evaluated using the log-rank test. Univariate logistic regression was used to compare categorical variables. Statistically significant variables (p < 0.05) from the univariate analysis were entered into a multivariate logistic regression model. Characteristics were selected a priori to adjust for potentially confounding variables, such as gender, maternal age, type of delivery, gestational size, gestational age, birthweight, admission respiratory distress, and admission level of consciousness. The case mix upon admission was adjusted by including the NMS, which predicts in-hospital mortality, in the final model. Data are reported as odds ratios (ORs) with 95% confidence intervals (CIs). The variables included in the final model were examined for multicollinearity, and no collinearity among variables was detected. All statistical analyses were two-sided, and P value less than 0.05 were considered significant. Statistical analyses were conducted using Stata 15 (StataCorp LP, College Station, TX).

### Sensitivity analysis

Sensitivity analyses were conducted to assess how the time of admission compares with the time of death, with two ways of defining daytime and overnight hours. Time of death reflects the cumulative care of neonates over days and nights. Among the neonates who died, the time of death was abstracted from the death certificate. Neonates without a death certificate were excluded from the sensitivity analysis. In the first sensitivity analysis, duty time was defined as overnight hours between 17:00 to 07:59 the following day, weekends (Saturday and Sunday), and public holidays according to the Ethiopian calendar. Working time was defined as daytime hours between 08:00 to 16:59 from Monday to Friday and not during public holidays according to the Ethiopian calendar. In the second sensitivity analysis, the time of death was analyzed exclusively between daytime and overnight hours, not accounting for weekends or public holidays. Finally, we explored how the acuity of neonates upon admission to the NICU influences survival by generating a Kaplan-Meier survival curve for neonates identified as high and low acuity according to the Neonatal Mortality Score.

## Results

Among the 812 infants admitted to the NICU during the study period, 342 (42%) were admitted during the day and 470 (58%) were admitted overnight. There were 207 deaths and 605 survivors. The median length of stay for those who died was 26 hours and the median length of stay for those who survived was 41 hours. There were 276 neonates identified with a high Neonatal Mortality Score above 12 and 536 neonates with a low Neonatal Mortality Score less than and equal to 12.

**Fig 1:**
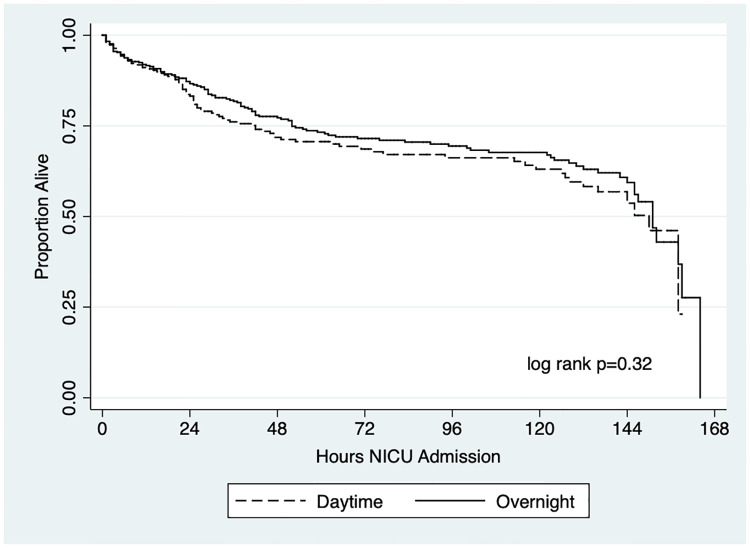
Kaplan-Meier survival curve comparing daytime and overnight NICU admissions.

Among the study population, the in-hospital mortality was similar among neonates admitted during overnight and daytime hours (26% vs 25%, respectively, p = 0.89). A Kaplan-Meier survival curve showed that the unadjusted time from NICU admission to death was comparable (Fig 1, log-rank p = 0.32). The median survival time (50% mortality) for neonates admitted during overnight and daytime shifts were 151 hours and 150 hours, respectively.

Table 2 shows the univariate analysis of perinatal characteristics and clinical variables. Gestational age, birthweight, maternal age, and parity were similar between the daytime and overnight groups. Additionally, there was no significant difference in the proportion of infants delivered by cesarean section between overnight and daytime admissions (30% vs 29%, respectively, p = 0.75). Infants admitted overnight versus daytime were more likely to be suctioned (22% vs 17%, respectively, p = 0.05) and receive bag-valve-mask ventilation at delivery (19% vs 14%, respectively, p = 0.03). Of admission vital signs, only the admission heart rate greater than 160 beats per minute was found to be significantly different between overnight and daytime admissions (8% vs 13%, respectively, p = 0.03). In assessing the case mix, there was no statistically significant difference among the neonates identified as high risk of mortality from the NMS between overnight and daytime admissions (34% vs 34%, p = 0.85). Furthermore, there were no differences in admission level of consciousness, admission respiratory distress, birthweight, or gestational age between daytime and overnight admissions. This analysis showed that the risk factors between overnight and daytime admissions cohorts were similar. The following variables were excluded from the analysis because more than 15% of the data were missing: duration of labor, rupture of membranes, and APGAR scores at 1 and 5 minutes.

**Table 2:** Comparison of characteristics admitted to the NICU during daytime and overnight hours.

Characteristic	Daytime (%) (n = 342)	Overnight (%) (n = 470)	OR	95% CI	P-value
Maternal age, years					0.82
<20	7%	8%	1.12	0.63–2.01	
21–29	65%	66%	1		
≥30	28%	26%	0.93	0.66–1.32	
Parity					0.51
1	52%	50%	1		
≥2	48%	50%	1.10	0.83–1.45	
Admission Age, hours					0.62
≤1	52%	54%	1		
>1	48%	46%	0.93	0.70–1.23	
Gender					0.82
Male	60%	60%	1		
Female	40%	40%	0.97	0.73–1.29	
Gestational Age, weeks					0.97
≥ 37	66%	65%	1		
32–36	23%	24%	1.03	0.74–1.44	
<32	11%	11%	1.04	0.65–1.64	
Birthweight, grams					0.93
≥2,500	57%	57%	1		
1,500–2,499	32%	33%	1.00	0.74–1.36	
<1,500	11%	10%	0.92	0.57–1.47	
Onset of Labor					0.90
Spontaneous	94%	94%	1		
Induced	6%	6%	0.96	0.50–1.84	
Duration of Labor					0.58
<24 hours	88%	89%	1		
≥ 24 hours	12%	11%	0.87	0.54–1.41	
Rupture of Membranes					0.68
< 24 hours	93%	92%	1		
≥ 24 hours	7%	8%	1.14	0.61–2.10	
Delivery					0.75
Vaginal	71%	70%	1		
C-section	29%	30%	1.05	0.77–1.43	
Antenatal Care (≥1)	93%	96%	1.62	0.89–2.96	0.12
APGAR Score 1 minute					0.84
< 7	41%	40%	0.96	0.67–1.38	
≥ 7	59%	60%	1		
APGAR Score 5 minute					0.84
< 7	18%	17%	0.95	0.60–1.51	
≥ 7	82%	83%	1		
Suctioned at Delivery	17%	22%	1.42	0.99–2.03	0.05
Bag & Mask at Delivery	14%	19%	1.51	1.03–2.21	0.03
Intubated at Delivery	3%	2%	0.72	0.31–1.68	0.45
CPAP on admission	23%	22%	0.92	0.66–1.29	0.64
Admission Heart Rate					0.03
<100	1%	2%	2.57	0.71–9.28	
100–160	86%	89%	1		
>160	13%	8%	0.61	0.39–0.96	
Admission Respiratory Rate					0.39
<35	6%	8%	1.18	0.67–2.07	
35–55	41%	44%	1		
>55	52%	48%	0.86	0.64–1.14	
Admission Temperature, Celsius					0.89
<36.0	65%	64%	0.89	0.61–1.31	
36.0–36.4	11%	11%	0.86	0.50–1.47	
36.5–37.5	16%	18%	1		
>37.5	7%	7%	0.81	0.43–1.50	
Admission Respiratory Distress					0.67
None	49%	49%	1		
Mild	8%	8%	1.07	0.62–1.82	
Moderate	30%	27%	0.90	0.65–1.25	
Severe	13%	16%	1.20	0.79–1.83	
Admission Level of Consciousness					0.50
Alert	84%	83%	1		
Irritable	2%	3%	1.85	0.71–4.83	
Lethargic	12%	11%	0.94	0.61–1.47	
Comatose	2%	3%	1.39	0.58–3.32	
Gestational Size					0.29
Small for Gestational Age	34%	32%	0.96	0.71–1.29	
Appropriate for Gestational Age	62%	62%	1		
Large for Gestational Age	4%	6%	1.62	0.84–3.12	
Head Size					0.63
Microcephalic	6%	8%	1.28	0.70–2.33	
Normocephalic	63%	60%	1		
Macrocephalic	31%	32%	1.11	0.80–1.54	
Neonatal Mortality Score					0.85
≤ 12 (low acuity)	66%	66%	1		
>12 (high acuity)	34%	34%	1.03	0.77–1.62	
Length of stay					0.08
≤ 24 hours	47%	41%	1		
>24 hours	53%	59%	1.28	0.97–1.70	

The multivariate analysis revealed that overnight admissions did not have increased odds of mortality with an adjusted OR of 1.02 (95% CI 0.64–1.62) (Table 3).

**Table 3:** Multivariate analysis comparing daytime and overnight NICU admissions.

Characteristic	ß coefficient	Adjusted OR^a^	95% CI	P-value
NICU Survival				
Survived	0	1		
Died	0.02	1.02	0.64–1.62	0.93
Gestational Age, weeks				
≥ 37	0	1		
32–36	0.03	1.03	0.63–1.68	0.91
<32	0.16	1.18	0.48–2.90	0.72
Birthweight, grams				
≥2,500	0	1		
1,500–2,499	0.16	1.17	0.69–1.99	0.56
<1,500	0.04	1.04	0.41–2.63	0.94
Delivery				
Vaginal	0	1		
C-section	0.04	1.04	0.74–1.45	0.84
Admission Heart Rate				
<100	0.84	2.32	0.59–9.04	0.23
100–160	0	1		
>160	-0.65	0.52	0.30–0.91	0.02
Admission Temperature, Celsius				
<36.0	-0.33	0.72	0.45–1.16	0.18
36.0–36.4	-0.18	0.84	0.45–1.57	0.58
36.5–37.5	0	1		
>37.5	0.006	1.0	0.47–2.18	0.99
Admission Respiratory Distress				
None	0	1		
Mild	-0.03	0.97	0.54–1.76	0.93
Moderate	-0.10	0.90	0.56–1.45	0.68
Severe	0.50	1.64	0.81–3.34	0.17
Admission Level of Consciousness				
Alert	0	1		
Irritable	0.94	2.55	0.77–8.44	0.13
Lethargic	-0.16	0.85	0.48–1.53	0.60
Comatose	-0.41	0.67	0.22–2.04	0.48
Gestational Size				
Small for Gestational Age	-0.07	0.93	0.62–1.39	0.72
Appropriate for Gestational Age	0	1		
Large for Gestational Age	0.44	1.56	0.72–3.36	0.26
Suctioned at Delivery	0.13	1.14	0.60–2.18	0.69
Bag & Mask at Delivery	0.30	1.36	0.67–2.74	0.40
Neonatal Mortality Score				
≤ 12 (low acuity)		1		
>12 (high acuity)	-0.15	0.86	0.46–1.61	0.64

^a^ Adjusted for gender, maternal age, and gestational size.

Intercept: 0.50, p = 0.54.

**Fig 2:**
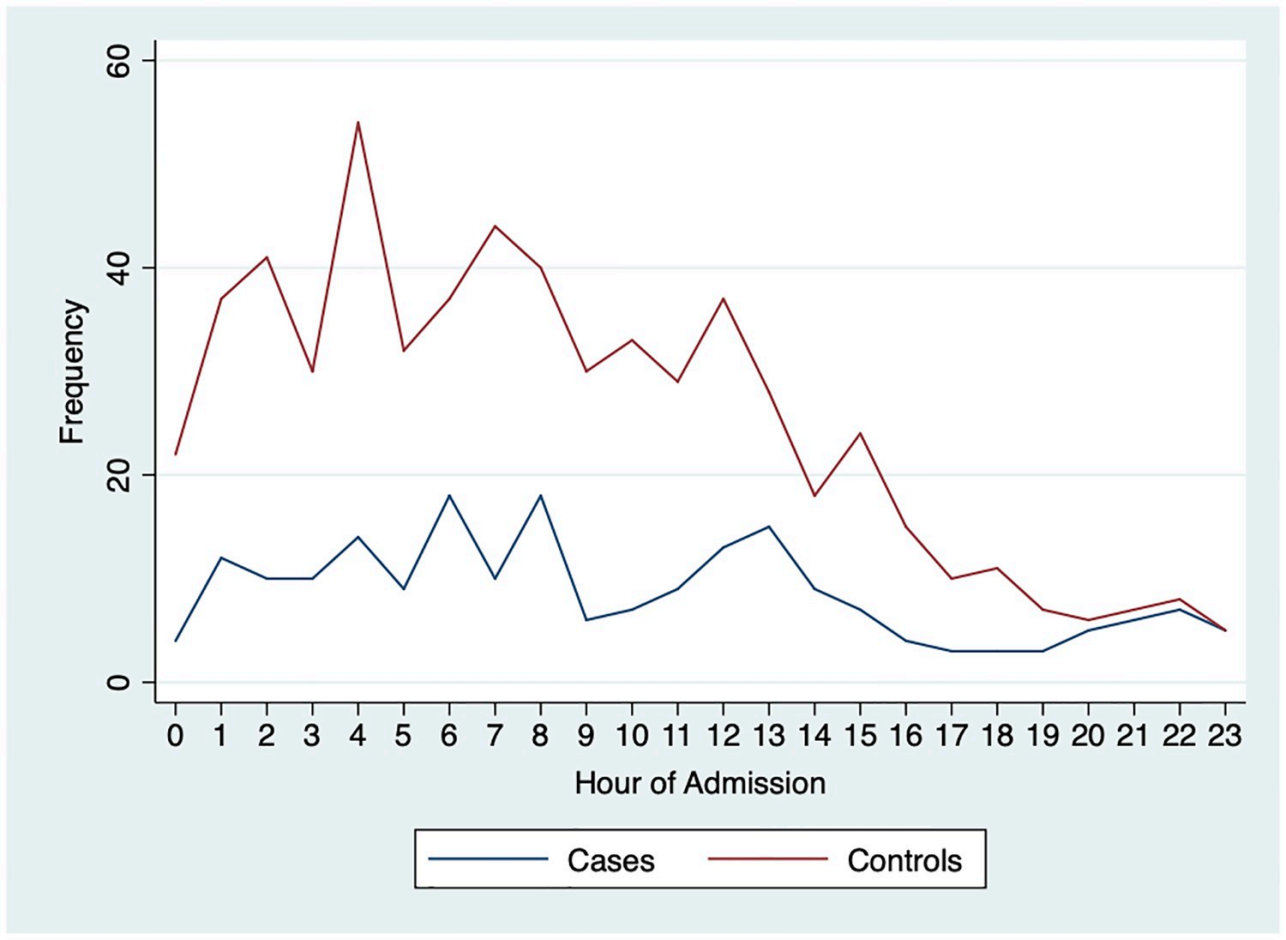
Overall admissions and neonatal deaths by time of NICU admission.

While there were more NICU admissions overnight than during the daytime, the number of deaths remained largely consistent (Fig 2). There were more admissions between midnight and 0900 and the number of admissions decreased starting in the late afternoon before increasing after midnight.

**Fig 3:**
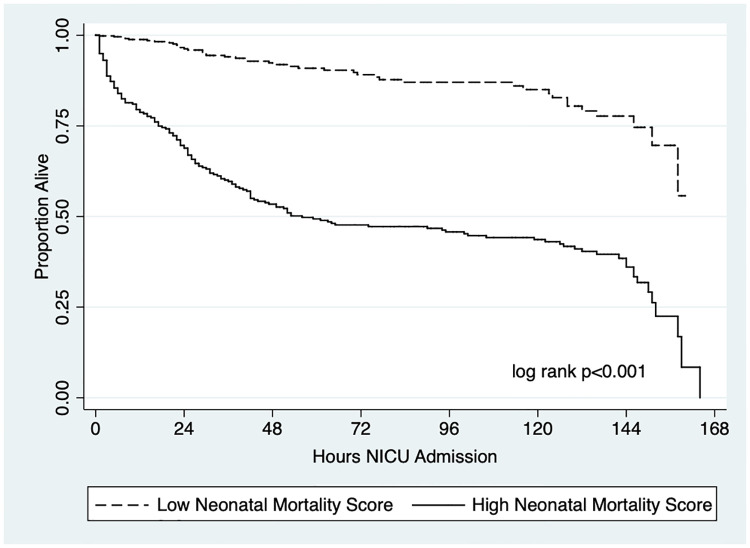
Kaplan-Meier survival curve comparing low and high neonatal mortality scores.

For the sensitivity analysis, 168 out of the 207 neonatal deaths in the study had a time of death reported in the chart. Among these 168 neonates, 129 deaths occurred overnight and during public holidays and weekends, and 116 deaths occurred overnight. The only difference among neonates who died overnight compared with neonates who died overnight and during public holidays and weekends was an increased odds of the admission heart rate being less than 100 beats per minute. Other admission vital signs and demographic and perinatal variables were similar among neonates who died during daytime and overnight hours. Lastly, a Kaplan-Meier survival curve showed that neonates with a low Neonatal Mortality Score survived longer than neonates with a high Neonatal Mortality Score (log-rank p<0.001, Fig 3).

## Discussion

Our study demonstrates that time of admission is not associated with clinical characteristics of the neonates, type of delivery, or in-hospital mortality among infants admitted to the NICU at the University of Gondar. Controlling for the acuity of illness, neonates in our study admitted overnight were no more likely to die than neonates admitted during the day. As a primary outcome, we analyzed the time of admission to the NICU because admission is arguably the more critical time period for seriously ill infants due to the need for stabilization and resuscitation [18]. In our sensitivity analysis of cases, we found that there was no significant difference in the severity of illness between neonates dying during working daytime and overnight hours. This reflects a consistent level of care during the time of admission, during the hospitalization, and the care prior to death.

These results stand in contrast to previous studies looking at the effect of admission time on neonatal mortality. In a study of a Canadian NICU network, researchers found that there was a 60% higher mortality rate among overnight admissions in infants born at <32 weeks’ gestation, equivalent to 29 deaths per 1,000 infants [11]. A study from Australia showed that infants born at night (defined as 18:00 to 08:00) had twice the risk of death compared to infants born during the day [12]. There is also literature suggesting that neonates born on weekends have a higher risk of dying than infants born on weekdays [19, 20]. It is important to note, however, that these studies looked at neonatal mortality trends only in high-income countries, which may not be generalizable to our study population. Additionally, the heterogeneity noted between these studies likely reflects the diverse delivery of care and organizational structure of the hospitals and ICUs where these studies were carried out.

We conducted a novel study examining the temporal relationship between NICU admission and mortality specifically in a low-income country. Determining whether a relationship exists between the time of admission and clinical characteristics and outcome of neonates is of particular importance because of the limited access to essential infrastructure and resources in Ethiopia and other low-income countries [7, 21]. One study out of Ethiopia found that only two-thirds of health facilities surveyed had the necessary equipment for neonatal resuscitation [21]. Furthermore, despite the fact that respiratory distress syndrome (RDS) accounts for almost half of the preterm mortality in the country, chest x-rays are performed infrequently and interventions specific to RDS, such as CPAP, are lacking [7].

There are several possible explanations for the lack of association between time of admission and mortality seen at the University of Gondar. Previous studies have shown that due to lower staffing levels, reduced access to diagnostic and therapeutic services, and fewer experienced providers during overnight shifts than during the day, overnight admissions could be associated with poorer outcomes [22]. In contrast, the results from our study may be reflective of a consistent staffing pattern across shifts which is adequate in limiting morbidity and mortality. Overnight, the University of Gondar NICU has one pediatrics resident dedicated to caring for premature neonates and one resident dedicated to term neonates. This resident staffing pattern may help explain the similar in-hospital NICU mortality between day and overnight admissions in the study. It has also been postulated in prior studies that a difference in case-mix may have played a role in the increased mortality overnight [11, 20].

More neonates with bradycardia upon NICU admission were admitted overnight, which has not been reported in other studies. Furthermore, the odds of admitting neonates with a mental status characterized by irritability increased overnight. Overnight, the odds decreased among neonates with tachycardia upon admission. Nonetheless, there were small numbers of neonates with these conditions, so these results may be influenced by the low sample size.

The acuity of neonates admitted to the NICU overnight versus during the day was not substantially different. However, in the sensitivity analysis, we found that neonates with higher Neonatal Mortality Scores had worse survival in comparison to neonates with lower Neonatal Mortality Scores, suggesting that acuity on presentation may play more of a role than the time of admission for in-hospital NICU mortality.

This study has several limitations. First, this is a retrospective study of prospectively collected data, which is susceptible to selection bias and residual confounding. Second, the results may only be generalized to tertiary care NICUs in Ethiopia at academic hospitals. Third, our analysis did not account for NICU-specific differences in physician coverage between daytime and overnight admissions. Our study did not capture neonatal mortality in terms of live births, so the resulting NMR cannot be compared to the overall NMR in Ethiopia. Finally, our study was not able to elucidate differences in how long infants received CPAP during daytime versus nighttime shifts, which could be useful for future studies.

Improving the quality of care of NICUs in low- and middle-income countries will be necessary to achieve substantial reductions in neonatal mortality. NICUs with more favorable health outcomes, like the one at the University of Gondar, can potentially serve as models for infrastructure and staffing in hospitals in LMICs. Further qualitative and implementation research are needed to understand contextual factors that have affected these data. From there, differences in staffing patterns and other factors influencing the quality of care can be compared across NICUs both in Ethiopia and in other LMICs.

## Supporting information

S1 Checklist STROBE statement—Checklist of items that should be included in reports of *case-control studies*. (DOC)

S1 Data (XLSX)
